# Network meta-analysis reaches nutrition research

**DOI:** 10.1007/s00394-018-1849-0

**Published:** 2018-10-31

**Authors:** Lukas Schwingshackl, Anette Buyken, Anna Chaimani

**Affiliations:** 1 Department of Epidemiology, German Institute of Human Nutrition Potsdam-Rehbruecke (DIfE), Arthur-Scheunert-Allee 114-116, 14558 Nuthetal, Germany; 2 NutriAct-Competence Cluster Nutrition Research Berlin-Potsdam, Berlin, Germany; 3 Public Health Nutrition, Institute of Nutrition, Consumption and Health, Faculty of Natural Sciences, University of Paderborn, 33098 Paderborn, Germany; 4 Paris Descartes University, Paris, France; 5 INSERM, UMR1153 Epidemiology and Statistics, Sorbonne Paris Cité Research Center (CRESS), METHODS Team, Paris, France; 6 Cochrane France, Paris, France

**Fig. 1:**
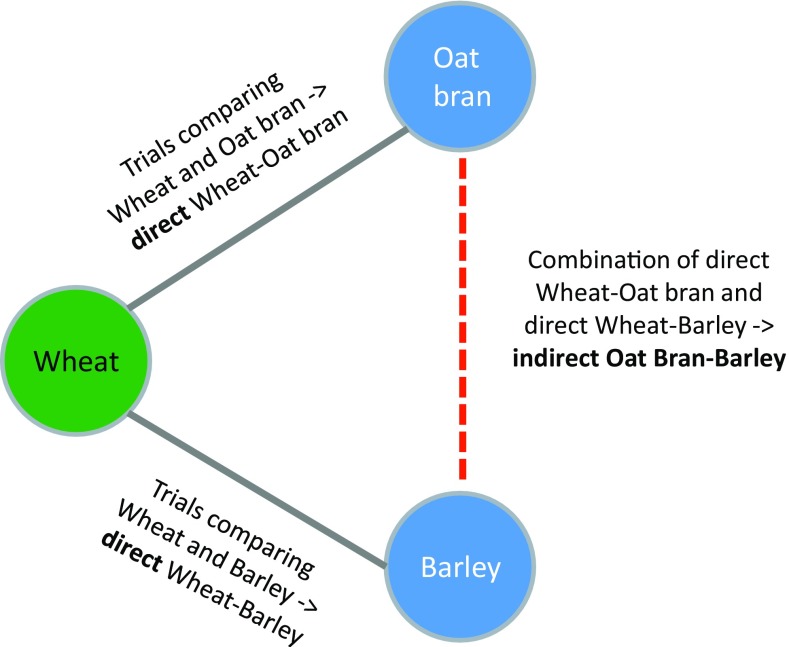
Example of indirect relative effects in a triangle comparing three interventions (wheat, oat bran, barley)

Network meta-analysis (NMA) is increasingly recognized as a promising evidence synthesis method commonly allowing stronger conclusions on the comparative effectiveness of healthcare interventions than conventional pairwise meta-analysis [1]. Its strength arises from the fact that it allows to synthesize both direct and indirect evidence from randomized trials. It is hence timely that Hui et al. recently published an NMA in the European Journal of Nutrition [2], comparing the effects of different whole grains (oat, brown rice, barley, and wheat) and brans (oat bran and wheat bran) on blood lipids (total cholesterol, LDL-C, HDL-C, and triacylglycerols), using data from 55 trials. NMA allows inference on every possible pairwise comparison of interventions within a connected network. For example, in the paper by Hui et al. [2], oat bran and barley have not been directly compared in a randomized trial, but each has been compared with wheat (Fig. 1). As such, an indirect comparison between oat bran and barley can be obtained. Sometimes, the relative effects estimated by the network may rely to a notable extent on indirect comparisons (i.e., for which no trials were ever conducted); the influence of direct and indirect evidence on the results can be seen using the contribution matrix [3, 4]. In fact, in the NMA by Hui et al., the contribution of direct evidence to the relative effects estimated by the network was very low ranging from 0.3% (oat vs. wheat) to 15.9% (wheat vs. control).

Nutrition research can substantially profit from the potential of NMA. However, it is crucial that authors meticulously plan, conduct, and report NMA [5, 6]; in particular, authors should follow a study protocol published *a priori* so as to improve transparency and perform a rigorous risk of bias assessment within and across studies as well as an evaluation of the quality of evidence. As Hui et al. are among the pioneers of applying NMA to the field of nutrition research, we draw on their article to highlight some methodological challenges that require specific attention when performing NMA.

Summary effects from NMA are usually presented in a league table including all comparisons: Hui et al. [2] identified oat bran as the most effective intervention strategy, revealing clinically relevant mean differences (MD) in comparison with the control diet [improvements in total cholesterol (TC) (MD: − 0.35 mmol/L, 95% CI − 0.47, − 0.23 mmol/L) and LDL-C (MD: − 0.32 mmol/L, 95% CI − 0.44, − 0.19 mmol/L)]. Another unique feature of NMA is its ability to rank interventions in relation with the studied outcomes, using the distribution of the ranking probabilities and the surface under the cumulative ranking curve (SUCRA) [7]. SUCRA ranges from 0%, i.e., the treatment always ranks last without uncertainty, to 100%, i.e., the treatment always ranks first without uncertainty. In the NMA by Hui et al. [2] oat bran ranked as the best treatment for TC (SUCRA: 97%), LDL-C (SUCRA: 97%), and triacylglycerols (TG) (SUCRA: 78%), followed by oat (SUCRA: 79% for TC, 64% for LDL-C, 76% for TG).

The extent to which NMA allows valid indirect inference depends on the extent to which the fundamental assumption of NMA usually called the ‘transitivity’, assumption is likely to be plausible. Transitivity requires that the trials comparing different sets of interventions are appreciably comparable in characteristics (other than the interventions being compared) which may affect the outcome [8, 9]. Transitivity should be evaluated prior to conducting NMA [8, 9], e.g., by examining whether the distributions of potential effect modifiers are comparable across the direct treatment comparisons. Transitivity would, for example, be violated if changes in body weight would differ strongly between the two direct comparisons, as depicted in Fig. 1. An important limitation of the NMA by Hui et al. is the lack of a formal investigation of the distributions of potential effect modifiers across the available direct comparisons.

They did, however, apply a number of approaches (loop-specific approach, node-splitting approach, and design-by-treatment interaction model) to examine statistical incoherence [2]. The coherence assumption suggests that direct and indirect evidence are in statistical agreement and its assessment is mandatory. Incoherence can be tested locally (i.e., in parts of the network) and globally (i.e., in the entire network) and the use of both types of tests is highly recommended [6]. If incoherence is identified, subgroup analyses and network meta-regression may be used to investigate potential sources. It should be noted that the absence of statistically significant incoherence—as in the case of Hui et al. [2]—is not necessarily evidence of the absence of incoherence. In particular in networks that built on scarce evidence—as in the NMA of Hui et al.—tests for incoherence have low power; thus, their results should be interpreted cautiously [10]. It is hence welcome that the authors performed several sensitivity analyses confirming the findings of their main analyses.

Addressing publication bias in NMA is as difficult as in pairwise meta-analysis; thus, priority must be given to the exhaustive search for unpublished studies. Assessment of small-study effects is usually the first step in the formal investigation of reporting biases [11]. Hui el al used the ‘comparison adjusted funnel plot’, a modified funnel plot for application to a network of trials [4]. Should funnel plot asymmetry be detected, meta-regression allows the estimation of the magnitude of small-study effects, while ‘selection models’ can investigate the potential for publication bias [12].

Hui and colleagues [2] also assessed the confidence of evidence, using the Grading of Recommendations Assessment, Development and Evaluation (GRADE) framework that considers the following items: study limitations, imprecision, inconsistency, indirectness, and publication bias. While the confidence on evidence was low for most comparisons, for comparisons of oat bran vs. the control diet it was judged as high for TC and moderate for LDL-C and TG. Hence, further research will provide important evidence on the majority of the comparisons included in this NMA, yet confidence is appreciable for oat bran vs. control diets. As this is an important conclusion for future research, we believe that a confidence on evidence statement should always be incorporated in the conclusion section of any manuscript. The use of the CINeMA (Confidence In Network Meta-Analysis: http://cinema.ispm.ch/) framework, which is an improvement of a previously suggested approach [3], can greatly facilitate judgements about the confidence that can be placed in results obtained by NMA. CINeMA modified and extended the five GRADE domains for use in NMA and is transparent and applicable to any network structure [3].

In view of its potentials, there is a risk that NMA is applied in cases, where the scarcity of available data precludes the estimation of precise results and the evaluation of its assumptions. Specifically, in the absence of direct evidence for several comparisons transitivity and incoherence cannot be formally tested, although transitivity can always be evaluated clinically and epidemiologically. In that case, the resulting relative effects would possibly be estimated with large uncertainty and the relative rankings might be meaningless.

In spite of these risks, we expect that high-quality NMAs combining the results of dietary intervention trials will become the new evidence synthesis norm also in nutrition research. While widely applied in many medical fields [13], its use in the field of nutrition is at present surprisingly rare. A quick search in PubMed (September 10th, 2018) using search-terms network meta-analysis[tiab] OR multiple treatments meta-analysis[tiab] OR mixed-treatment comparison[tiab] AND (diet*[tiab] OR nutrition[tiab]) yielded only 38 hits. Out of these, only ~ 50% original NMA papers dealt with a nutrition-related topic and only two of them [14, 15] were published in a nutrition journal. Yet, NMAs have the potential to advance the knowledge in the field of nutrition as they provide insights that cannot be obtained by individual trials or pairwise meta-analysis: for example, the DASH dietary approach proved to be the most effective dietary approach to reduce blood pressure among hypertensive and pre-hypertensive patients [15], while the Mediterranean diet emerged as the most effective dietary approach to improve glycaemic control in type 2 diabetes patients [16]. Finally, butter and lard were ranked worst for reducing LDL-C, whereas safflower-, rapeseed-, and sunflower oil performed best [17].

Beyond this, NMA has recently been proposed also as a tool to plan the optimal design and the required sample size of new trials [18]. Finally, NMA may be used to close the gap between evidence stemming from meta-analyses of prospective observational studies and missing evidence from RCTs. Using dietary exposures comparable to those examined in observational studies on hard clinical endpoints in NMAs on intervention trials with intermediate disease markers could ultimately strengthen the credibility of nutrition research findings [19, 20].
